# Feature-Level Fusion of Surface Electromyography for Activity Monitoring

**DOI:** 10.3390/s18020614

**Published:** 2018-02-17

**Authors:** Xugang Xi, Minyan Tang, Zhizeng Luo

**Affiliations:** School of Automation, Hangzhou Dianzi University, Hangzhou 310018, China; xixi@hdu.edu.cn (X.X.); 11045404@hdu.edu.cn (M.T.)

**Keywords:** surface electromyography (sEMG), feature-level fusion, monitoring, Davies–Bouldin Index (DBI), support vector machine (SVM)

## Abstract

Surface electromyography (sEMG) signals are commonly used in activity monitoring and rehabilitation applications as they reflect effectively the motor intentions of users. However, real-time sEMG signals are non-stationary and vary to a large extent within the time frame of signals. Although previous studies have focused on the issues, their results have not been satisfactory. Therefore, we present a new method of conducting feature-level fusion to obtain a new feature space for sEMG signals. Eight activities of daily life (ADLs), including falls, were performed to obtain raw data from EMG signals from the lower limb. A feature set combining the time domain, time–frequency domain, and entropy domain was applied to the raw data to establish an initial feature space. A new projection method, the weighting genetic algorithm for GCCA (WGA-GCCA), was introduced to obtain the final feature space. Different tests were carried out to evaluate the performance of the new feature space. The new feature space created with the WGA-GCCA effectively reduced the dimensions and selected the best feature vectors dynamically while improving monotonicity. The Davies–Bouldin index (DBI) based on fuzzy c-means algorithms of the space obtained the lowest value compared with several fusion methods. It also achieved the highest accuracy when applied to support vector machine classifier.

## 1. Introduction

As the concept of healthcare shifted from disease diagnosis and treatment to disease prevention, the importance of technical development in systematic and continuous exercise management has been emphasized [1]. As a consequence of the growth of the aging population, the number of elderly or frail people who need help in their daily activities is rapidly increasing [2,3,4]. This change has led to the focus on the importance of activity monitoring. To name a few, Mantilla quantitatively determined the reliability of longitudinal diaphragm EMG analyses and provided an important tool for evaluating the progression of diseases or injuries that impair ventilation [5]; the effects of shoulder flexion/extension combined with elbow flexion angle on discomfort score were investigated by Farooq for repetitive gripping task [6]. Also, the expansion of activity tracking and personal data collection offers the potential for patient engagement in the management of chronic diseases. Consumer wearable devices for activity tracking also have shown promise in post-surgery recovery in cardiac patients, pulmonary rehabilitation, and activity counseling in diabetic patients, among others [7].

With the rapid development of wireless networks and wearable sensing technologies, advances in microelectromechanical systems and information and communications technology have created new opportunities for automatic activity monitoring, which can improve the quality of life of the elderly and frail people and provide them with adequate medical services [8]. Wireless and wearable sensors, such as accelerator sensors, inertial sensors, and gyroscopes, have been widely used in numerous studies.

Electromyography (EMG) sensors are widely applied in medical diagnosis, rehabilitation, and human–computer interaction [9,10,11]. Compared with other wearable sensors, EMG sensors can directly indicate the human body’s electrophysiological responses to various activities. EMG detects which muscles are recruited for an action, generates information about the behavior being performed, and determines the effort required to perform such behavior [12]. In the behavior monitoring context, this ability of EMG has significant advantages for classifying behavior [13]. Specifically, EMG has the inherent advantages of distinguishing between passive and active activities, predicting movements, and achieving a short calculating time [14]. Many existing technologies use EMG together with mechano-myographic sensors to acquire signals that can be used to control prosthetic devices, thus enabling people with disabilities to train and restore control over their residual muscles [15]. For example, Betthauser et al. presented a sparsity-based adaptive classification method for the EMG, which aims to achieve a good performance in the offline and online contexts involving untrained upper-limb positions for amputees and able-bodied subjects [16].

Nevertheless, in the search for the best feature set from original datasets, considerable challenges continue to exist [17]. This difficulty is due to the bioelectrical of EMG signals; hence, they can be influenced by many disturbing factors, such as electrode displacement, postural changes, and individual-dependent features such as condition of muscles, subcutaneous fat, skin surface, etc. [18]. At the same time, EMG is sensitive to external factors, such as experimental setting, recording site, or natural environment. Another realistic problem is that EMG involves an excessive number of inputs. Different feature vectors extracted from the same pattern always reflect different characteristics of patterns. By optimizing and combining these different features, effective discriminant information of multiple features is retained, and redundant information is eliminated to a certain degree [19]. Thus, extensive efforts need to be done to obtain optimal feature sets before their application to classifiers. Liu investigated the effects of feature dimensionality reduction strategies on the classification of surface EMG (sEMG) and employed a Markov random field method and a forward orthogonal search algorithm to evaluate the contribution of each individual feature to the classification [20]. Buchenrieder combined selection and projection and applied them in the time and frequency domain [21]. A cross-correlation-based feature extraction technique for EMG signals was proposed in [22], and the results of support vector machine (SVM) and k-nearest neighbor were evaluated to have a high classification accuracy and increased efficiency and robustness.

Feature fusion has been increasingly used in the field of pattern recognition as a result of the richness of nature records in the database. Feature fusion typically comprises three levels: pixel-level fusion, feature-level fusion, and decision-level fusion. Pixel-level fusion refers to the fusion at the original data layer. Decision-level fusion combines results from different methods, algorithms, sources, or classifiers to generate estimates that are of better quality than those obtained from any individual source alone [23]. Feature-level fusion is the most important pre-processing step for any classifier.

The main goal of feature fusion is to utilize all relevant lower dimensional features extracted from the same or different modality profiles of objects for classification. However, the extracted features (or signals) may contain redundant and irrelevant information, which increases the dimensionality and degrades the quality of feature spaces. In such a case, conventional data integration techniques fail to provide proper interpretation. In addition, dimensionality increases the learning parameters of classifiers and thus increases the calculation complexity and the memory requirement. Thus, dimensionality reduction by feature mapping is needed.

Feature mapping or feature projection, as the most widely used method for feature fusion, can be described as mapping from an original feature space to an appropriate subspace such that a learning criterion is optimized. Feature projection enables high-dimensional feature vectors to be visualized in a low dimension and allows the distribution of the reduced feature vectors to be analyzed [24]. The canonical correlation analysis (CCA) method is developed in feature fusion to extract the feature pairs depicting the intrinsic correlation between two sets of features [19]. CCA finds the correlation between two sets of multidimensional vectors extracted from raw data, and then maximizes the correlation in the transformed space to seek a pair of projection vectors (PVs). As a result, CCA provides an effective strategic tool to extract intrinsic sets of features by integrating two views of the same object for viable clinical application [25], particularly for the classification of normal and pathological EMG signals [26]. The identified features after fusion are especially robust in real-life applications, especially when the best feature sets are unknown. Therefore, many scholars have begun to study the correlation of multiset variables.

In the current work, we propose a feature-level fusion technique, the weighting genetic algorithm for GCCA (WGA-GCCA). Firstly, the original feature is directly extracted by self-defined feature sets. Secondly, global canonical correlation analysis (GCCA) is applied to the feature sets to obtain the generalized canonical projective vector (GCPV) of the projected space. Thirdly, GA was used to select better projective vectors from GCPV. After weighing the projective vectors, it was used to create the final feature space.

The feature space obtained with the proposed method exhibits good characteristics in terms of dimensionality reduction. Apart from evaluating the performance of the feature space in the EMG region using the Davies–Bouldin index (DBI) of fuzzy c-means, we also investigate the accuracy and characteristics of monotonicity by SVM.

## 2. Subjects and Experiment Protocol

**Figure 1:**
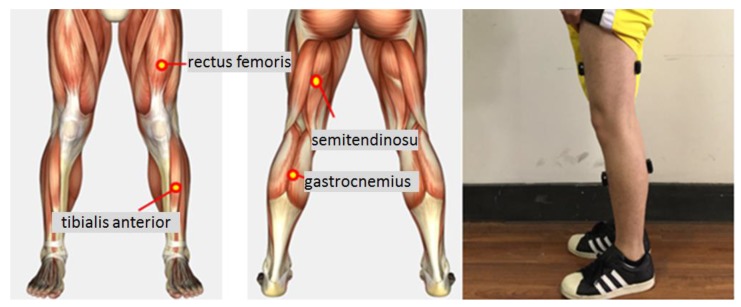
Muscles in Schematic and real placement.

**Figure 2:**
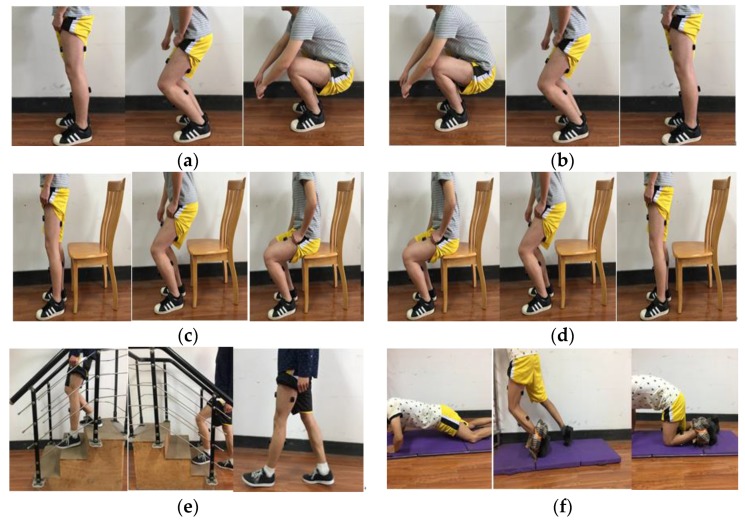
Eight activities of daily living. (**a**) stand-to-squat; (**b**) squat-to-stand; (**c**) stand-to-sit; (**d**) sit-to-stand; (**e**) walking, stair-ascending and stair-descending; (**f**) trip-fall.

Eight healthy, able-bodied subjects (six males and two females: age, 24–26 years; height, 160–180 cm; weight 46–75 kg) participated in the experiments. Each subject wore sensors on their gastrocnemius, rectus femoris, tibialis anterior, and semitendinosus from left lower limbs, shown in Figure 1, and performed eight ADLs: stand-to-squat (st–sq), squat-to-stand (sq–st), stand-to-sit (st–si), sit-to-stand (si–st), stair-ascending (a–s), stair-descending (d–s), walking (w), and falling (f). The participants repeated the procedure six times in each experiment day; the total number of repetition of each activity should be at least 240. Each behavior lasted for nearly two seconds, and the sampling frequency of sensors were set as 1000 Hz. The whole duration time was used for feature computation later. Experimental process was shown in Figure 2. Figure 2f was trip-fall, the ankle of the participant was fixed by a sponge ring, which limits the ankle movement and also plays an important role in protecting the ankle.

Our experiments were carried out in an air-conditioned laboratory to prevent heat from causing severe sweating for the participants. Each participant wore athletic shorts during the experiments, and the sensors were fixed to parts of the skin that were exposed to air so as to prevent physical contact or pressure. To keep the skin surface free from oil and dust, we scrubbed the skin with alcohol wipes before placing the sensors. Every half an hour, we scrubbed the skin again and replaced the sensors in the same place to prevent the influence of secretions. As for the daily activities, a large amount of data is needed. Hence, completing all the activities in one day is easy for causing muscle fatigue. Nevertheless, muscle condition varies in different days. Thus, we opted to have the participants complete a set of movements (eight ADLs) in a period of time. After each action, the participants were given 5 min of rest time; they were also given an appropriate rest time of 15 min after each round. We ensured the completion of three rounds in the morning and in the afternoon. We also aimed to complete the data collection within five days. In this way, we were able to collect the required data.

**Figure 3:**
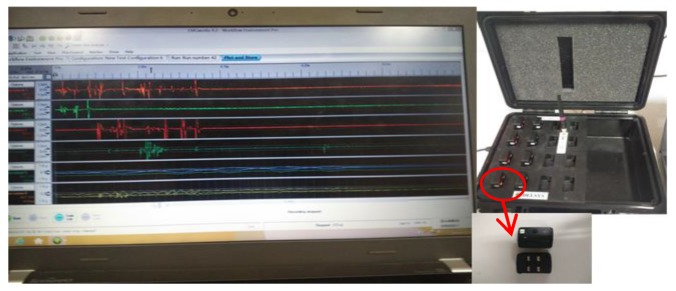
Delsys Full Wireless Surface Electromyography Test System (Trigno™ Wireless EMG).

Sensors we used in this study are Trigno™ Wireless EMG (Delsys Inc, Natick, MA, USA). It has 8-channel surface electromyography plus 24-channel acceleration acquisition. It is a sensor with motion artifact suppression (patent) that can be freely moved, the sensor directly transmits data wirelessly. It provided a 16-bit resolution, a bandwidth of 20–450 Hz, and a baseline noise <1.25 µV (rms) and has a typical operating range of 40 m and the communication protocol is Bluetooth. The sEMG signals were sampled at 1000 Hz using EMG works 4.0 (Delsys Inc, Natick, MA, USA), as show in Figure 3.

## 3. Feature Extraction

### 3.1. Feature Extraction

In machine learning, feature extraction starts from an initial set of measured data and builds the derived values (features) that are intended to be informative and non-redundant [27]. In this way, the process facilitates the subsequent learning and generalization steps and even improves human interpretations in some cases [27]. Feature extraction is generally divided into the time domain, frequency domain, time–frequency domain, and sometimes information entropy. A single time domain feature does not offer enough features for the system to recognize activities properly. Moreover, the time domain method suffers from poor robustness. Frequency domain methods are typically used in studies on muscle fatigue. Entropy spectrum features have a robust feature set for recognition systems of EMG [28]. The time–frequency domain characterizes varying frequency information at different time locations, thereby providing plenty of non-stationary information about the analyzed signals [29]. Thus, a combination of several feature extraction methods is considered to provide an effective feature set.

In our study, we created a feature set consisting of Wilson amplitude (WAMP), fuzzy entropy (FE), permutation entropy (PE), auto-regressive coefficient (ARCU) from third-order cumulant, and energy of wavelet coefficient (EWT). These methods have been found to be effective in [29].

### 3.2. New Feature Space

Feature sets obtained from the previous session would be a complex high-dimensional space. The ordinary method to turn complexity to simplicity is projection [30], and the most commonly used method is CCA. CCA is used to measure the linear relationship between two multidimensional variables. It aims to find a new set of bases that are optimal with respect to correlation and measures the corresponding correlations.

In our study, we aim to obtain an optimal feature space from the feature set mentioned above. This feature space should be based on, but better than, CCA.

#### 3.2.1. Global Canonical Correlation Analysis (GCCA)

GCCA is an extension of CCA and considers a class matrix in the correlation analysis.

Let $S_{W_{x}}$ and $S_{W_{y}}$ denote the within-class scatter matrix of samples *X* and *Y*, respectively

(1) $$S_{W_{x}}=\sum_{i=1}^{n}\sum_{j=1}^{n}{\left(x_{ij}-m_{x_{i}}\right)}^{T}(x_{ij}-m_{x_{i}})$$

(2) $$S_{W_{y}}=\sum_{i=1}^{n}\sum_{j=1}^{n}{\left(y_{ij}-m_{y_{i}}\right)}^{T}(y_{ij}-m_{y_{i}})\;$$

where $x_{ij}\in X,y_{ij}\in Y$ is the *j*-th training sample of class *i*, *n* is the number of training samples in class I, and $m_{x_{i}},m_{y_{i}}$ denote the mean vectors of samples *x* and *y* in class *i*, respectively.

The between-set covariance matrix of *X* and *Y* is given by

(3) $$S_{b_{xy}}=\frac{1}{n}\sum_{i=1}^{n}(x_{i}-m_{x}){\left(y_{i}-m_{y}\right)}^{T}$$

On the basis of the introduction above, we obtain the generalized canonical correlation discriminant criterion as

(4) $$J(x,y)=\frac{u^{T}S_{b_{xy}}v}{\sqrt{u^{T}S_{W_{x}}u•v^{T}S_{W_{y}}v}}$$

where u and v are pair vectors that maximize J(x,y) and are regarded as the projective directions. The combined features that are extracted using the linear transformation, which are composed of GCPV, are called the generalized canonical discriminant features.

(5) $$X^{*}={\left(u_{1}^{T}x,u_{2}^{T}x,\ldots u_{d}^{T}x\right)}^{T}={\left(u_{1},u_{2},\ldots u_{d}\right)}^{T}x=U^{T}x,$$

(6) $$Y^{*}={\left(v_{1}^{T}y,v_{2}^{T}y,\ldots v_{d}^{T}y\right)}^{T}={\left(v_{1},v_{2},\ldots v_{d}\right)}^{T}y=V^{T}y$$

The following two linear transformations will form the combined features projected, which will then be used for classification. *W*_1_ and *W*_2_ are named type I and type II in this paper.

(7) $$W_{1}=[X^{*}Y^{*}]=[U^{T}xV^{T}y]=\left[U^{T}\;V^{T}\right]\left[\begin{array}{cc} x & 0\\ 0 & y \end{array}\right]$$

(8) $$W_{2}=X^{*}+Y^{*}=U^{T}x+V^{T}y=\left[U^{T}\;V^{T}\right]\;\left[\begin{array}{c} x\\ y \end{array}\right]$$

#### 3.2.2. Weighting Genetic Algorithm of GCCA (WGA-GCCA)

GCCA combined the feature samples into one-plane including the information of class matrices, which resulted in a high-dimensional eigenvector. High dimensionality not only increases the complexity of the calculation but also causes the peaking phenomenon. This means that the classifier performance begins to degrade after a certain number of features. In such a case, feature reduction is needed.

The application of feature selection methods to our dataset of EMG features is expected to reduce dimensionality and aid in finding reliable subsets [31]. Our aim is to reduce the dimensionality of GCPV and thus make a second projection to gain a new feature space. Feature reduction methods were considered here.

Genetic algorithm (GA) belongs to a large class of evolutionary algorithms, which is a metaheuristic algorithm inspired by the process of natural selection. Compared with commonly used methods like principal component analysis (PCA) that uses an orthogonal transformation to produce uncorrelated and relevant features [32], and singular value decomposition (SVD) that factorizes a single matrix into three matrices, GA has an automatic learning process aimed at achieving good feature group selection. It is commonly used to generate high-quality solutions to optimization and search problems by relying on bio-inspired operators, such as mutation, crossover, and selection [33].

In the present work, the chromosomes of GA are composed of the features extracted in Table 1. Binary coding is used as the coding method of the genetic algorithm. The initial population is generated randomly. Fisher function value is used as the fitness.

Finally, we weighted each feature vector in a new space. General weighting methods include using the degree of separation of feature sets as basis. However, this type of weighting is not conducive for each feature to play their respective proportions. Thus, we adopted the dynamic weighting method based on clustering. We utilized the rates of clustering classifiers as the weight of each channel. The formulas are given by

(9) $$W_{i}=accu_{i}-accu_{min}+W_{min}$$

(10) $$r_{i}=\frac{W_{i}}{\sum_{i=1}^{n}W_{i}}$$

where $accu_{i}$ and $W_{i}$ are the recognition rate and weight of the *i*-th feature set, respectively; $accu_{min}$ and $W_{min}$ are the recognition rate and weight of the feature set with a minimum recognition rate, respectively; and $r_{i}$ is the weight coefficient of the *i*-th feature set.

**Figure 4:**
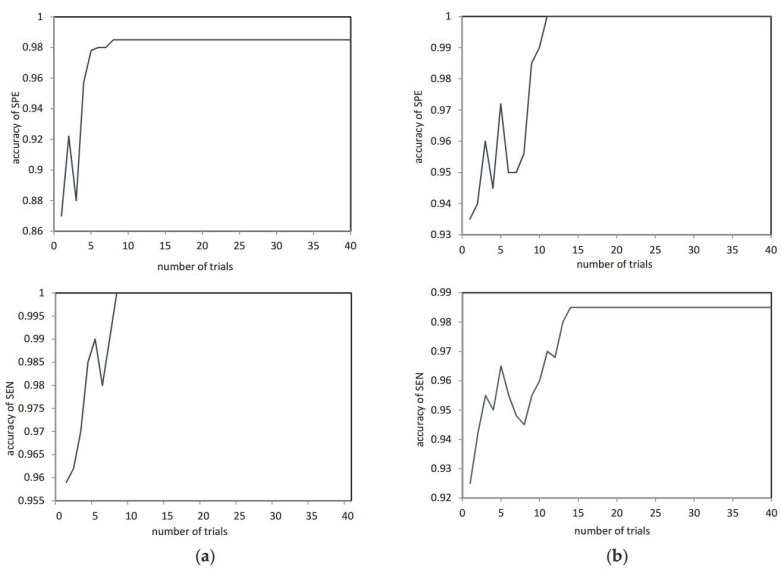
The SEN and SPE of WGA-GCCA with trial times in two examples. (**a**) One example result. (**b**) Another example result.

The additional processes in GA can cause severe computational cost, and thus crossover rate and mutation rate based on evolutionary algebra are used. The evolutionary algebraic decreasing function is

(11) $$x=\cos(\frac{gen}{gen\_\max}\cdot \frac{\pi }{2})$$

where gen and gen_max represent the current evolutionary algebra and total evolution algebra, respectively. Figure 4 depicts the sensitivity and specificity of the WGA-GCCA with trial times in two examples, showing the sensitivity (SEN) and specificity (SPE) can reach the highest value in the 15th iteration.

## 4. Classification and Results

**Figure 5:**
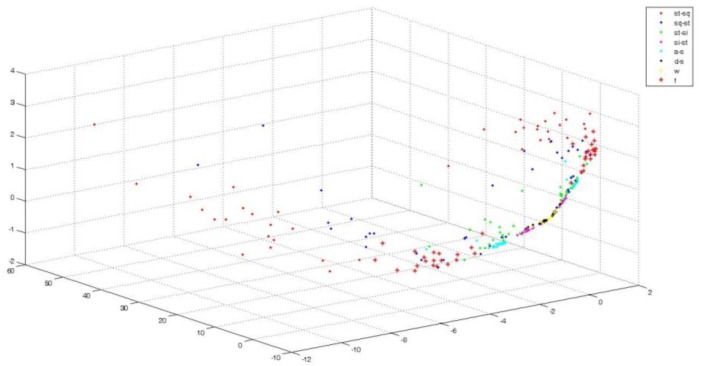
Three-dimensional projection figure of gastrocnemius after WGA-GCCA.

Figure 5 represents the three-dimensional projection figure of the sEMG of gastrocnemius after WGA-GCCA. The ADLs after projection showed a general distinction, especially for the sharp ones, such as stand–squat (st–sq), squat–stand (sq–st), and fall (f).

However, the evaluation of the performance of the proposed mapping strategy and fusion method for EMG signal fusion is not a straightforward task due to the many factors that characterize performance. Therefore, we first evaluated the performance of the new feature space using the DBI. Thereafter, we evaluated the classification performance together with accuracy, complexity, and monotonicity. Accuracy was evaluated with a kernel–SVM classifier to obtain the best recognition rates. Complexity was evaluated by comparing the obtained feature space with the GCCA feature space. Monotonicity was evaluated by adding muscle channel datasets, with the channel numbers ranging from one to six.

### 4.1. Davies–Bouldin Index (DBI) of New Feature Space

The DBI is a non-fuzzy clustering validity index. It is mainly based on the geometrical principle, which is rooted in the degree of discrepancy between clusters and the degree of data of objects located in the same class [34].

The DBI formula is given by

(12) $$DBI=\frac{1}{K}\sum_{i=1}^{K}max\{\frac{S_{i}+S_{j}}{d_{i.j}}\}$$

where *S_i_* represents the degree of data object dispersion in the i-th class; similarly, *S_j_* represents the degree of data object dispersion in the *j*-th class; *d_i,j_* represents the distance between the *i*-th and the *j*-th class; *K* denotes the number of clusters. Thus, a small DBI equates to a good feature space.

The DBI is typically used with the help of clustering methods. Thus, to evaluate the feature space we obtained, we adopted a fuzzy clustering method. Clustering is an unsupervised classification method that is used when the only data available are unlabeled and no structural information about such data is available [35]. C-means algorithms comprise one of the well-known partitional clustering methods that produce minimum squared-error partitions [36]. In the current work, we employed fuzzy c-means clustering (FCM). This is a clustering algorithm that uses membership degrees to determine the extent to which each data point belongs to a cluster. It divides the n vectors $x_{i}$ (*i* = 1, 2, ..., *n*) into c-fuzzy groups and finds the clustering centers of each group to minimize the value function of the non-similarity index.

**Figure 6:**
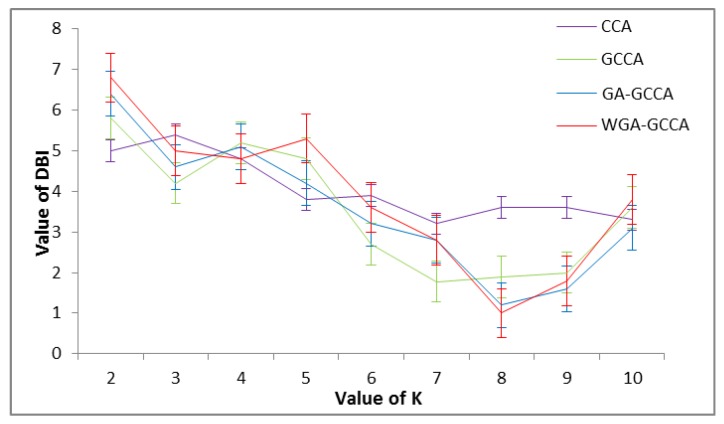
DBI with various numbers of clusters of CCA, GCCA, GA-GCCA, and WGA-GCCA.

The value of K varies in the range of 2–10. The variation of the DBI with the number of clusters for the above-mentioned feature spaces is shown in Figure 6. The figure shows that the DBI peaks at 8 for WGA-CCA and GA-GCCA; hence, these two feature spaces have better characteristics than GCCA and CCA. WGA-GCCA also exhibits a large variance, which means that the feature space can be classified into correct clusters effectively.

### 4.2. Accuracy

Accuracy improvement is essential for fusion method. We selected four muscle signals as inputs to evaluate accuracy performance. The four EMG channels were from gastrocnemius, tibialis anterior, rectus femoris, and semitendinosus. Four fusion methods—namely, CCA, GCCA, GA-GCCA, and WGA-GCCA—were then applied. The fused feature space was inputted into the classifier of kernel–SVM for recognition.

Kernel–SVM uses kernel methods in the support vector machine (SVM) models. Radial basis function (RBF) was used as the kernel function, given by

(13) $$K(x,x_{i})=\exp\text{\{}-\frac{{\left|x-x_{i}\right|}^{2}}{\sigma^{2}}\text{\}}$$

where σ is the width of nucleus, key to the effectiveness of classification. Particle swarm optimization (PSO) was utilized to seek optimum parameters in RBF, which has already been proved to be efficient in several existing studies [37,38].

From Section 2, at least 240 signals for each activity were collected, from which we chose 180 comparably clear signals, and six-fold cross validation was considered. The data sets were partitioned into six equal sized subsets, making the number of data for each set being 30. The first subset was used for calculating accuracy after using the remaining subsets as the training data. This process was repeated for each subset, and then averaged over six results. Table 2 shows the details of SEN (sensitivity) and SPE (specificity) of each method for the eight ADLs.

The table shows that type I is usually better than type II, as the former has more inputs to form a feature set. For each daily activity recognition, WGA-GCCA resulted in the best performance among all other methods.

Considering many popular feature selection techniques, such as the aforementioned PCA and SVD, this paper also introduced PCA-GCCA and SVD-GCCA. Generally speaking, GA based methods are computationally expensive. Table 3 thus shows not only the SEN and SPE of three selection-GCCA fusion methods, but also the calculation time.

In terms of the calculation time, SVD-GCCA fusion method has the lowest computational cost, as we chose to retain 1/4 the number of dimensions after GCCA. However, SEN and SPE of SVD-GCCA were comparably low. WGA-GCCA has a relatively slow calculating speed, but it has a high SEN and SPE. For off-line recognition, a recognition time of 0.5 s can be acceptable; for on-line recognition such as the use in self-rehabilitation robots, it can work efficiently as long as a self-run-up time is allowed.

To gain a clear result of the distinction of ADLs using WGA-GCCA, we form a confusion matrix as shown in Table 4, which indicates the frequency of correct and misclassified actions. The results showed that the fall action had the best SEN and SPE because the raw signal underwent an abrupt change. Some activities, such as stand-to-sit and sit-to-stand, are not easily distinguished each other, which make the SEN and SPE relatively low. For the gait action, WGA-GCCA SVM exerted a relatively satisfactory recognition effect. This result is due to the cyclical behavior of gait in the raw signals, which made this action different from the conversion action. For gait action, which utilizes different muscle weights, the dynamic weighting method used for it can be used employed for the action of specific gravity weighting. Thus, the identification of ADLs, especially gait recognition, offers a significant advantage.

### 4.3. Complexity

**Figure 7:**
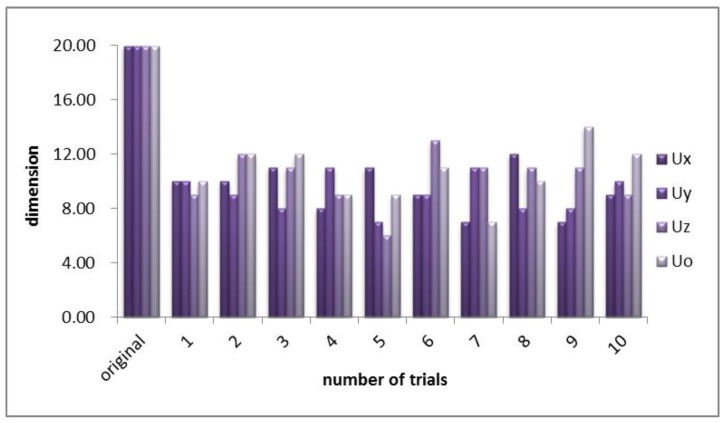
Dimension of feature sets after GA-GCCA fusion compared with GCCA. U*n* (*n* = x, y, z, o) Denotes the feature samples; x, y, z, o presents the four channels of data inputs.

Complexity, especially space complexity, affects the total amount of memory space of a classifier. Space complexity can be explained as a dimension in a sense. The feature space projected by GCCA expanded to four sets of 20-dimensional feature combinations, as it did not override the concern that the system needs to deal with relatively high dimensional data, as is often implicated in many practical applications of use-centered computing schemes, in which computation resources are limited [20]. To take into account the computational resource issue, we used feature selection to obtain few feature vectors while retaining the good features. For GCCA and GA-GCCA (WGA-GCCA), the recognition rates showed a similar trend. However, GA-GCCA and WGA-GCCA achieved feature selection and a good combination with minimal feature inputs. Figure 7 shows the dimension of feature sets after GA-GCCA fusion.

For the feature space after GCCA, the dimension was extended to 20 dimensions, as indicated by the original coordinate item. GA was then applied to achieve the dimensionality reduction effect. Although the GA is random, the test results showed that the overall dimension has been reduced to about half of the original; hence, the recognition rate is ensured (as described above).

### 4.4. Monotonicity

**Figure 8:**
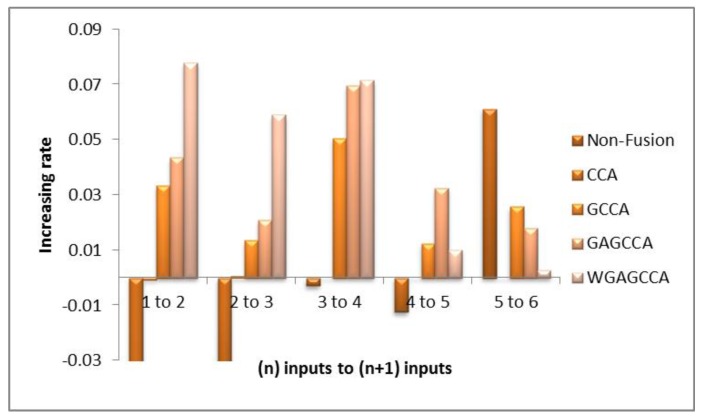
Increasing rate of recognition as the input increased from *n* to *n* + 1 (*n* = 1, 2, 3, 4, 5).

Monotonicity reflects in the improvement of results when adding a new feature descriptor. To verify the monotonic experiment, we added two sets of EMG signals from the medial femoral and lateral muscles. We tested varying numbers of muscle sets, from one muscle to six muscles, and get their recognition rates. Each EMG signal was pre-produced using the extraction method. Table 5 shows the average recognition rates of different numbers of sets of EMG signals by the SVM classifier. The features obtained without using fusion (non-fusion) and those obtained with the CCA fusion method showed no significant optimization when the number of inputs increased; on the contrary, the recognition rate decreased in a sense. Fusion methods based on GCCA exhibit obvious monotonicity (Figure 8). The figure shows the increasing rate of recognition as the input increased from *n* to *n* + 1 (*n* = 1, 2, 3, 4, 5). As can be seen, the increasing rate of WGA-GCCA is the highest among all groups, especially the input muscle number, which increased from one muscle to two muscles. Both the table and figure prove that WGA-GCCA has the best monotonicity.

## 5. Discussion

The purpose of this work is to solve the shortcoming of large inputs and the weakness of EMG signals and ultimately provide a reference for daily activity monitoring. Feature projection is a widely used feature-level fusion method. However, many similar methods have been used in image processing. Feature projection typically exploits the correlations between various data dimensions with the goal of creating dimensions/axes that are uncorrelated and sufficiently describe the data [39]. Thus, using feature projection in the EMG region makes sense, as it makes full use of corresponding muscles and thus reduces the effects of external factors on a single channel. The growth of data dimensionality poses challenges to many existing mining and learning algorithms. Feature selection has been proven to be an effective and efficient way to prepare high-dimensional data for data mining and machine learning [39]. We used GA as our selection method due to their automatic learning process.

This method could further be used in other regions or for a combination of different signals. This idea helps to achieve a high recognition rate and reliability for the development of activity monitoring systems.

## 6. Conclusions

A feature-level fusion method based on GCCA to detect surface EMG for activity monitoring is proposed, studied, and experimentally evaluated. The GCPV created by GCCA is optimum and selected by the GA to form a new feature space. The new feature space exhibits low dimensions and good monotonicity. The results also show that the feature space has the lowest DBI of the c-means cluster and the highest recognition rate of SVM.

This study, however, has several limitations and thus needs further development. First, the method presented did not reduce the calculation time because the GA selection method generally requires long calculations. Moreover, we are not certain about how well this algorithm would work in a real scenario with unscripted free-form activities performed by elderly or frail patients. Further experiments and studies will be performed to use the method in clinical assistive devices, walking assistance devices, and robotics or prosthetic devices.

## Figures and Tables

**Table 1:** Feature extractions in the initial feature set.

Extraction Type	Dimension of Single Inputs	Domain
WAMP	1	Time domain
FE	1	Entropy domain
PE	1	Entropy domain
ARCU	4	Time domain
EWT	5	Time–frequency domain

**Table 2:** SEN (sensitivity) and SPE (specificity) of different fusion methods for the eight ADLs (%).

			St–Sq	Sq–St	St–Si	Si–St	S–A	S–D	W	Fall
CCA	Type I	SEN	67.22	70.64	67.22	67.22	67.22	67.22	67.22	70.22
		SPE	72.50	69.50	69.50	69.50	69.50	69.50	69.50	69.50
	Type II	SEN	54.00	67.22	64.32	64.32	64.32	64.32	64.32	67.22
		SPE	67.22	87.50	54.00	54.00	54.00	54.00	54.00	64.32
GCCA	Type I	SEN	88.43	88.43	88.43	88.43	88.43	88.43	88.43	88.43
		SPE	92.11	92.11	92.11	92.11	92.11	92.11	92.11	92.11
	Type II	SEN	88.25	88.25	88.25	88.25	94.59	94.59	94.59	94.59
		SPE	87.50	87.50	87.50	93.33	93.33	93.33	93.33	67.22
GA-GCCA	Type I	SEN	95.91	95.91	95.91	94.59	94.59	94.59	94.59	97.22
		SPE	87.50	87.50	94.59	98.59	89.74	93.33	97.22	98.50
	Type II	SEN	90.91	90.91	91.35	91.35	91.35	91.35	91.35	95.91
		SPE	87.50	87.50	87.50	90.91	87.50	87.50	89.74	91.35
WGA-GCCA	Type I	SEN	97.50	96.60	98.50	100	98.50	100	100	100
		SPE	98.59	95.91	97.59	98.50	95.91	97.22	100	100
	Type II	SEN	95.50	95.91	90.91	97.50	90.91	100	100	100
		SPE	97.22	90.91	94.59	98.59	90.91	95.91	98.50	100

**Table 3:** Sensitivity (SEN), specificity (SPE), and calculation time (time) of the eight ADLs (%).

		St–Sq	Sq–St	St–Si	Si–St	S–A	S–D	W	Fall
WGA-GCCA	SEN (%)	97.50	96.60	98.50	100	98.50	100	100	100
	SPE (%)	98.59	95.91	97.59	98.50	95.91	97.22	100	100
	Time (s)	0.5091	0.5290	0.5123	0.4850	0.5264	0.5004	0.5133	0. 4505
PCA-GCCA	SEN (%)	78.75	86.25	90.88	91.25	92.35	84.35	90.88	82.56
	SPE (%)	80.02	92.32	86.25	86.78	88.75	81.25	87.5	80.00
	Time (s)	0.1361	0.1396	0.1369	0.1380	0.1431	0.1245	0.1227	0.1311
SVD-GCCA	SEN (%)	75.50	73.75	0.6802	86.25	86.25	83.75	87.25	72.34
	SPE (%)	78.29	76.68	0.6000	82.36	81.75	79.62	82.75	76.25
	Time (s)	0.0834	0.0879	0.0880	0.0881	0.0834	0.0903	0.0871	0.0991

**Table 4:** Confusion matrix of ADLs for WGA-GCCA (%).

	St-Sq	Sq-St	St-Si	Si-St	S-A	S-D	W	Fall
St–Sq	97.5 ± 2.5	1.5 ± 2.0	0	0	0	0	0	1.0 ± 0.5
Sq–St	2.1 ± 1.3	96.6 ± 3.3	1.3 ± 1.0	0	0	0	0	0
St–Si	0	0	98.5 ± 1.5	1.5 ± 1.5	0	0	0	0
Si–St	0	0	0	100 ± 0	0	0	0	0
S–A	0	0	0	0	98.5 ± 1.5	1.5 ± 1.5	0	0
S–D	0	0	0	0	0	100 ± 0	0	0
W	0	0	0	0	0	0	100 ± 0	0
Fall	0	0	0	0	0	0	0	100

**Table 5:** Accuracy when different numbers of inputs are used in the SVM classifier.

	One	Two	Three	Four	Five	Six
Non-Fusion	0.6280	0.5577	0.5229	0.5214	0.5149	0.5464
CCA	0.5	0.4996	0.5	0.5	0.5	0.5
GCCA	0.7661	0.7917	0.8024	0.8429	0.8534	0.8754
GA-GCCA	0.7845	0.8186	0.8357	0.8738	0.8929	0.9095
WGA-GCCA	0.7845	0.8456	0.8655	0.9095	0.9392	0.9719
